# Altered Gut Microbiota Associated With Hemorrhage in Chronic Radiation Proctitis

**DOI:** 10.3389/fonc.2021.637265

**Published:** 2021-10-15

**Authors:** Liangzhe Liu, Chaoyun Chen, Xia Liu, Bingcheng Chen, Chen Ding, Jinjun Liang

**Affiliations:** ^1^ Center for Clinical Precision Pharmacy, The First Affiliated Hospital of Guangdong Pharmaceutical University, Guangzhou, China; ^2^ School of Clinical Pharmacy, Guangdong Pharmaceutical University, Guangzhou, China; ^3^ Department of Biomedical Science, City University of Hong Kong, Hong Kong, Hong Kong, SAR China; ^4^ Department of Colorectal Surgery, The Affiliated TCM Hospital of Guangzhou Medical University, Guangzhou, China; ^5^ School of Clinical Integrative Chinese and Western Medicine, Guangzhou Medical University, Guangzhou, China; ^6^ Department of Surgery, Maoming Hospital of Traditional Chinese Medicine, Maoming, China; ^7^ Department of Electrical Engineering, City University of Hong Kong, Hong Kong, Hong Kong, SAR China

**Keywords:** chronic radiation proctitis, hematochezia, gut microbiota, *Peptostreptococcaceae*, *Lachnospiraceae*

## Abstract

Pelvic cancer radiotherapy may cause chronic radiation proctitis (CRP) that adversely affects patient’s quality of life, especially in patients with prolonged hematochezia. However, previous studies of radiation enteropathy mainly focused on acute irradiation hazards, and the detailed pathogenesis process and mechanism of prolonged hematochezia associated with radiation-induced toxicity remain unclear. In this study, we characterized the gut microbiota of 32 female CRP patients with or without hematochezia. Differential patterns of dysbiosis were observed. The abundance of *Peptostreptococcaceae*, *Eubacterium*, and *Allisonella* was significantly higher in CRP patients with hematochezia, while the compositions of the *Lachnospiraceae*, *Megasphera*, *Megamonas*, and *Ruminococcaceae* were lower in the microbiota of non-hematochezia patients. Functional prediction suggested significant difference in the expression of mineral absorption and the arachidonic acid metabolism proteins between hematochezia and non-hematochezia patients, possibly interdependent on radiation-induced inflammation. This study provides new insight into the altered composition and function of gut microbiota in patients with hematochezia, implying the potential use of probiotics and prebiotics for assessment and treatment of CRP.

## Introduction

Pelvic radiotherapy is one of the most important treatments of gynecologic tumors. Chronic radiation proctitis (CRP) is a well-known complication of pelvic radiotherapy that occurs in 5% to 20% of the cancer survivors 6 months to several years later (1). Unlike acute radiation enteropathy (RE), which presents with acute diarrhea and requires immediate termination of radiotherapy (2), CRP commonly manifests hematochezia, which adversely affects patient’s quality of life in the long term. Prolonged, recurrent rectal inflammatory and bleeding lead to weakness, anemia, and a series of mental effects including anxiety and depression, especially in female patients (3, 4). Current treatments of CRP including high doses of 5-amin-osalicylic acid enemas, endoscopic thermal formalin therapy, and argon plasma coagulation rely on medical equipment and professional physicians (5–7). Development of daily oral drugs for improving CRP symptoms with better compliance are under the spotlight (8).

With the deepening understanding of intestinal homeostasis, prebiotics and probiotics have been proposed to be the future treatment option of an array of chronic diseases. The significance of the liver–microbiome axis has been recognized as a major modulator of liver diseases (9, 10). Also, gut microbial dysbiosis has been reported to play crucial roles in a series of gastrointestinal disorders including inflammatory bowel diseases (IBD), irritable bowel syndrome (IBS), and RE. A multi-omics study of the gut microbial ecosystem described both host and microbial activities with characteristic taxonomic that caused functional and biochemical shifts in IBD (11). DNA fingerprinting and cloning-sequencing techniques revealed that acute postradiotherapy diarrhea might be associated with patient’s initial microbial composition (12). Higher counts of *Clostridium* IV, *Roseburia*, and *Phascolarctobacterium* are significantly associated with RE (13). *Lactobacillus rhamnosus* GG (LGG) protection of intestinal epithelium from radiation injury was illustrated by a rodent model. The LGG-mediated radioprotection is reported to be dependent on MyD88, TLR-2, and COX-2 (14). However, little is known about the interaction between gut microbiota and prolonged proctorrhagia after irradiation.

In the present study, we set out to characterize the gut microbiota profiles of CRP patients and demonstrated the specific dysbiosis in CRP patients with hematochezia.

## Materials and Methods

### Ethics Statement and Patient Information

This study was approved by the Ethics Board at Guangzhou Hospital of TCM. Samples were collected from 32 female outpatients under a proctoscope. Informed consent was obtained from all subjects. The patients received pelvic radiotherapy 1 to 2 years prior to the onset of chronic radiation proctitis. No antibiotic has been adopted by any patient for at least 3 months. Thirteen patients complained of hematochezia for at least three times per day, and bleeding was observed under a proctoscope. These patients were classified as the H group. Sixteen patients who complained of diarrhea with no or little blood in stool were classified as the NH group (**Table 1**). Samples from three healthy volunteers were collected in parallel. Vegetarian, patients complicated with irritable bowel disease or genetic hemorrhagic disease, and patients who have received antibiotics or steroid treatment in the past 3 months were excluded from this study.

**Table 1:** Clinical characteristics of the patients.

Characteristics	NH (*n* = 16)	H (*n* = 13)	*p*-value
Age (years)	58.5 (18.25)	57 (22)	0.26
Female	16 (100%)	13 (100%)	1
Type of malignancy			1
Cervix cancer	14 (87.5%)	12 (92.3%)	
Uterus cancer	2 (12.5%)	1 (7.7%)	
Hemafecia frequency (times per month)	2 (1.875)	30 (39)	0.020621
Erosive mucosa and rectal bleeding	0 (0)	13 (100%)	0
DRE, fresh blood on glove	1 (6.3%)	13 (100%)	<0.0001

Results presented as frequency (%) or median (interquartile range). DRE: digital rectal examination.

### DNA Extraction

Total DNA was extracted from a 0.25-g sample by a QIAamp PowerFecal DNA Kit (QIAGEN, DE). The DNA concentration and purity were determined by Multiskan™ GO (Thermo Fisher Scientific, US), and the integrity of the DNA was determined by agarose gel electrophoresis.

### 16S rRNA Gene Sequencing

PCR was performed on the V4 region (515F-806R) of the 16S rRNA gene of the sample bacteria by priming different indexes at both ends. The reaction system of PCR was performed using 10 μl of KAPA HiFi HotStart ReadyMix (KAPA Biosystems, USA), 2 μl of DNA (30 ng/μl), and 1 μl of forward and reverse primer (10 μM). The forward primer of 16S was 5’-gtgccagcmgccgcggtaa-3’ and the reverse primer was 5’-ggactacnvgggtwtctaat-3’. The following thermal cycling program was used: an initial denaturation step at 95°C for 3 min followed by 30 cycles of 95°C for 20 s, 60°C for 30 s (annealing/synthesis step), 72°C for 30 s (extension), and finally 72°C for a 10-min extension (9, 12, 15). The PCR products were purified with the AxyPrep™ PCR Cleanup Kit (Axygen, USA), and the concentration was determined with Qubit 3.0 (Thermo Fisher Scientific, USA). Equal amounts of each sample were mixed together to form a sequencing library. QSEP100 (Bioptic, CHN) and ABI7300 quantitative PCR (Thermo Fisher Scientific, USA) were used to detect and quantify the insertion fragment and concentration of the library, respectively. The tested libraries were Paired-End 150 bp (PE150) sequenced using the Illumina MiniSeq platform (16).

### Bioinformatic Analysis

Sequences generated from Illumina sequencing were analyzed with MOTHUR (version 1.39.5) for data cleaning and chimera removal, identification of operational taxonomic units (OTU), taxonomic assignment, and community comparison by adapting its standard operational procedure (17). Sequences were realigned with the SILVA-compatible alignment database (http://www.mothur.org/w/images/9/98/Silva.bacteria.zip). The 3% dissimilarity cutoff value was used for assigning an OTU. Shannon’s diversity, Simpson’s diversity, ACE, and Chao I richness indices were generated with the MOTHUR program. Output matrixes were further analyzed by principal coordinates analysis (PcoA) based on UniFrac distance (18, 19) and linear discriminant analysis (LDA) effect size (LEfSe) to identify differences in relative abundance at different taxonomic levels (20). Gene function and metabolic pathway prediction was conducted by PICRUSt (19).

### Statistics and Data Visualization

The analysis of variance (ANOVA) test was used to assess the differences in the similarity index and the number of bands among the three groups: controls, patients with hematochezia, and patients without hematochezia. The Mann–Whitney *U* test was used when we analyzed difference between two groups using GraphPad prism 8 (21). Krona was used for visualization of microbiome community (22).

## Results

### Patients and Sample Collection

Thirty-two female participants were assigned into two groups: group NH, meaning chronic radiative proctitis (CRP) patients with no or mild hematochezia (hematochezia frequency < twice per week and/or no fresh blood observed under proctoscope, *n* = 16); and group H, meaning CRP patients with hematochezia (hematochezia frequency > twice per week and/or fresh blood was found under proctoscope, *n* = 13) (**Table 1**). Three patients were excluded as they presented with rectal ulcers (diameter > 1 mm) and/or symptoms of bacterial infection. The age of the 29 patients ranged from 28 to 71 years, and no significant difference was found between the two groups. Patients have received a total dose of 48 to 54 Gy irradiation as for radiotherapy of uterus or cervical carcinoma but have not received antibiotic treatment for at least 3 months. Frequency of uterus or cervix cancer was similar between two groups. Three samples from healthy adults were collected and analyzed in parallel.

### α- Diversity of Gut Microbiota in CRP Patients

**Figure 1:**
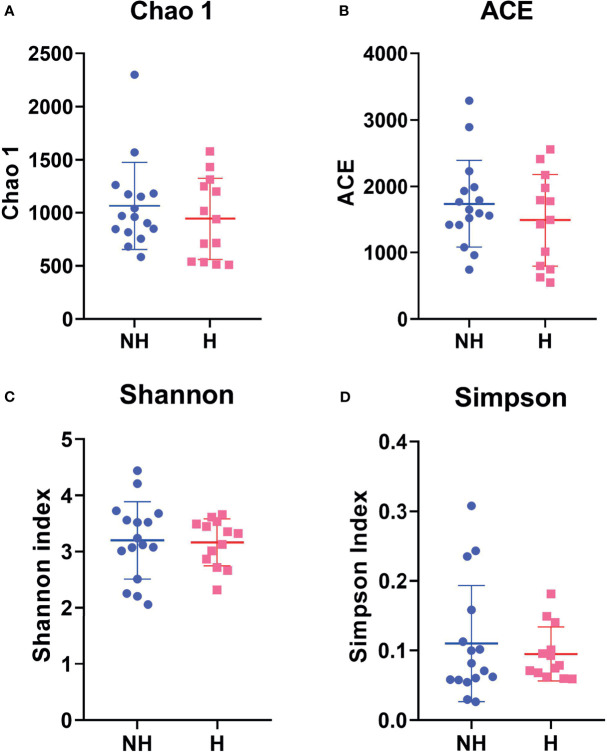
Comparative α-diversity analysis by **(A)** Chao1, **(B)** ACE estimator, **(C)** Shannon, and **(D)** Simpson Index indicating microbiome differences between non-hematochezia (NH) and hematochezia (H) subjects. Error bars indicate SEM.

To investigate the ecological complexity of the gut microbiota of CRP patients, α-diversity analysis within each group was conducted using Community richness calculators Chao1 estimator and ACE estimator as well as Community diversity calculators the Simpson index and the Shannon index in MOTHUR. As shown in **Figure 1**, the Community richness in CRP patients with hematochezia (Mean of Chao1 = 1065; Mean of ACE = 1738) as compared with CRP patients without hematochezia (Mean of Chao1 = 943.0; Mean of ACE = 1,488) was not statistically different (*p* > 0.1). Neither Community richness in healthy volunteer was distinguished (**Supplementary Figure S1**). This result suggests that the overall complexity of gut microbiota may not be a sensitive indicator for CRP.

### Altered β-diversity of Gut Microbiota in CRP Patients

**Figure 2:**
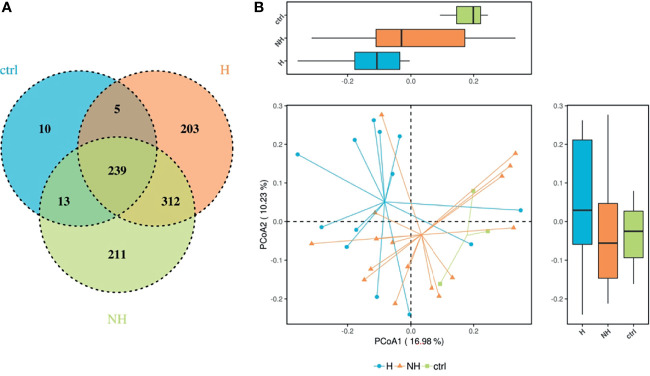
Differential composition of gut microbiome in CRP patients. **(A)** Venn diagram representing the operational taxonomic units (OTUs) in samples obtained in healthy volunteers (crtl), CRP patients with hematochezia (H), and those without hematochezia (NH). **(B)** PCoA based on UniFrac matrix showing the differential microbiota composition. Boxes represent the 25th to 75th percentile of the distribution; the median is shown as a thick line in the box.

The 16S rRNA gene amplicon data contained 1,005 OTUs, 133 families, and 342 genera. Venn diagram was generated to illustrate the assigned OTUs in each group. About 70% of the OTUs were shared in CRP patients while 27% and 29% of the taxonomic units were unique in the H group and NH group, respectively (**Figure 2A**). Ordination analysis including Principal Component Analysis (PCA), Principal Co-ordinates Analysis (PCoA), and Non-Metric Multi-Dimensional Scaling (NMDS) were conducted to measure the difference in bacterial community composition in two dimensions. The first principal component (PC1) of PCA and PCoA largely distinguished changes among healthy controls and CRP patients with hematochezia (**Figure 2B** and **Supplementary Figure S2**). Inter-individual variation accounted for part of the variance, which was consistent with previous studies (9, 11). In addition, the heatmaps demonstrate differential composition of microbiota in three levels (**Supplementary Figure S3**). The β-diversity of gut microbiota in CRP patients suggests that a proportion of bacteria population is related to hematochezia.

### Different Bacterial Taxa Between the Hematochezia and Non-Hematochezia Group

**Figure 3:**
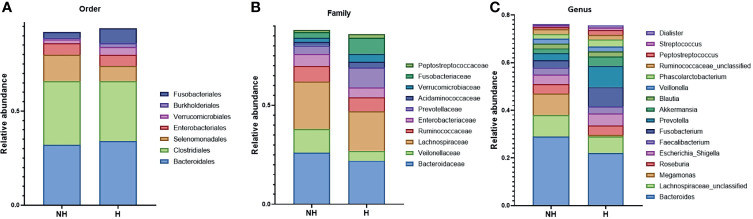
The relative abundance of the bacterial taxa with more than 2% of the abundance. **(A)** The seven most abundant bacteria in the order level. **(B)** The 10 most abundant bacteria in the family level. **(C)** The 16 most abundant bacteria in the genus level.

To further study the effect of microbiota composition on CPR symptom, the OTU matrixes were clustered into three cohorts. Hematochezia and non-hematochezia groups were isolated and visualized by Krona (**Supplementary Figure S4**). Taxonomy of the filtered bacteria with more than 2% of total abundance is shown in **Figure 3**. Changed compositions of more than twofold were observed in *Fusobacteriales* and *Selenomonadales* orders; *Peptostreptococcaceae*, *Fusobacteriaceae*, *Verrucomicrobiaceae*, *Prevotellaceace*, and *Veilonelaceae* families; and *Peptostreptococcus, Akkermansia, Prevotella*, and *Megamonas* genera.

**Figure 4:**
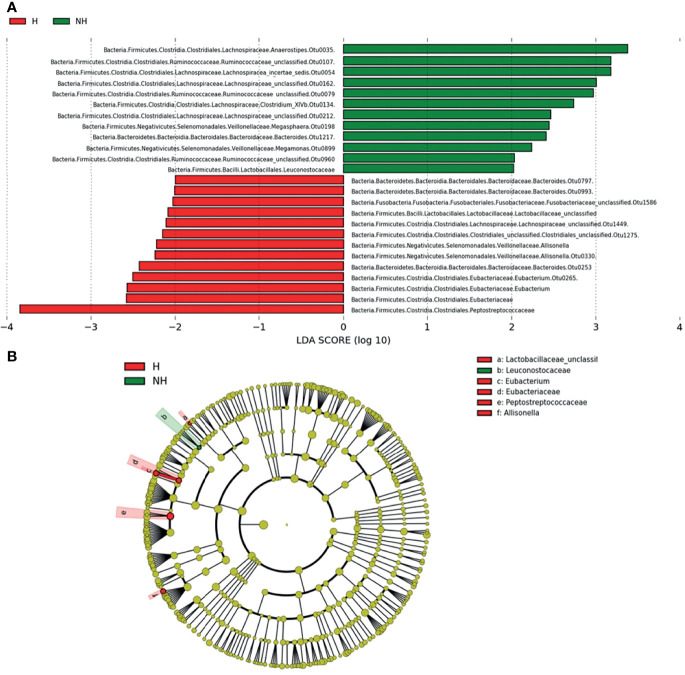
LDA score **(A)** and evolutionary diagram **(B)**, with circles representing taxonomic levels from phylum to genus/species. Each small circle at a different classification level represents a classification at that level, and the diameter of the small circle is proportional to the relative abundance. The species with no significant difference were yellow, while the differential species were colored as indicated.

With the linear discriminant analysis (LDA) on effective size (LefSe), we generated a global view of gut microbiota and revealed 25 bacteria taxa with differential relative abundance in both NH and H groups (LDA score > 2, **Figure 4**). The LDA result was consistent with the Krona plots. The *Peptostreptococcaceae, Eubacterium*, and *Allisonella* were enriched from the hematochezia group, while the *Ruminococcaceae, Megasphera*, and *Megamonas* were distinguished from the non-hematochezia group. Dominant population of *Bacteroidaceae* and *Lachnospiraceae* in each group depend on species. OTU 35, 54, 162, 134, and 212 of *Lachnospiraceae* were significant in the non-hematochezia group. OTU 797 and 993 of *Bacteroidaceae* were found in the hematochezia group.

**Figure 5:**
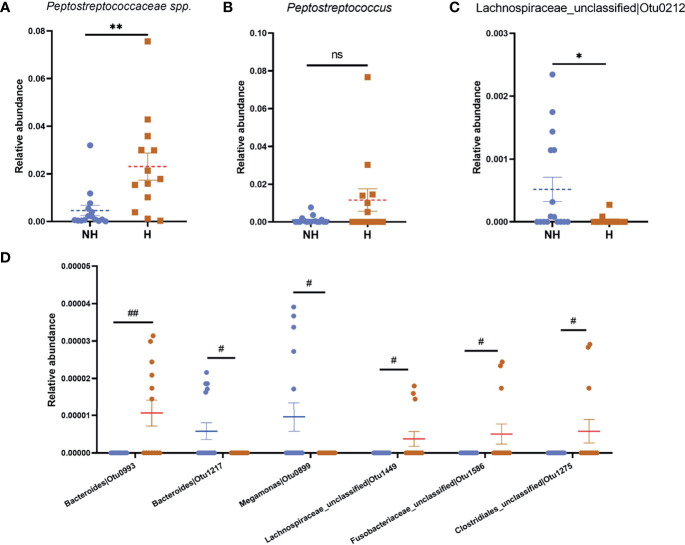
Significantly differential relative abundance of bacteria in Heamafecia patients. The relative abundance of Peptostreptococcaceae species **(A)**, *Peptostreptococcus* OTU45 **(B)** and *Lachnospiraceae* OTU212 **(C)** are shown. 4 OTU were unique in H group while 2 OTU were found in NH group **(D)**. Error bars indicate SEM. Blue dots: Relative abundance of bacteria in NH; brown squares: Relative abundance of bacteria in H group. ns, not significant, *p < 0.05, **p < 0.01, ^#^qFDR < 0.1, ^##^qFDR < 0.05.

*Peptostreptococcus* is the most abundant genus within the *Peptostreptococcaceae* family (accounts for >70% of the family in the H group; 84% in the NH group, **Supplementary Figure S4**), which met the highest LDA score in the hematochezia group. We compared the relative abundance of *Peptostreptococcus* OTU45 from each group and found that the data distribution was similar with that of *Peptostreptococcaceae* (**Figure 5**), which indicated that the abundance of *Peptostreptococcus* contributed to dysbiosis in hematochezia patients. In contrast, the relative abundance of *Lachnospiraceae* OTU212 was significantly higher in the non-hematochezia group, occupying 0.1% of the rectal microbiome. In addition, *Bacteroidaceae* OTU993, *Lachnospiraceae* OTU1449, *Fusobacteriaceae* OTU1586, and *Clostridiales* OTU1275 were uniquely found from the hematochezia group. *Bacteroidaceae* OTU217 and *Megamonas* OTU899 were identified from the non-hematochezia group (**Figure 5**, **Supplementary Figure S5**).

These data imply that the bacterial taxa in the hematochezia group is interfered and may alter the microenvironment in colorectum, leading to aggravation of chronic radiation proctitis symptoms.

### Microbial Functional Dysbiosis in the Hematochezia Group

**Figure 6:**
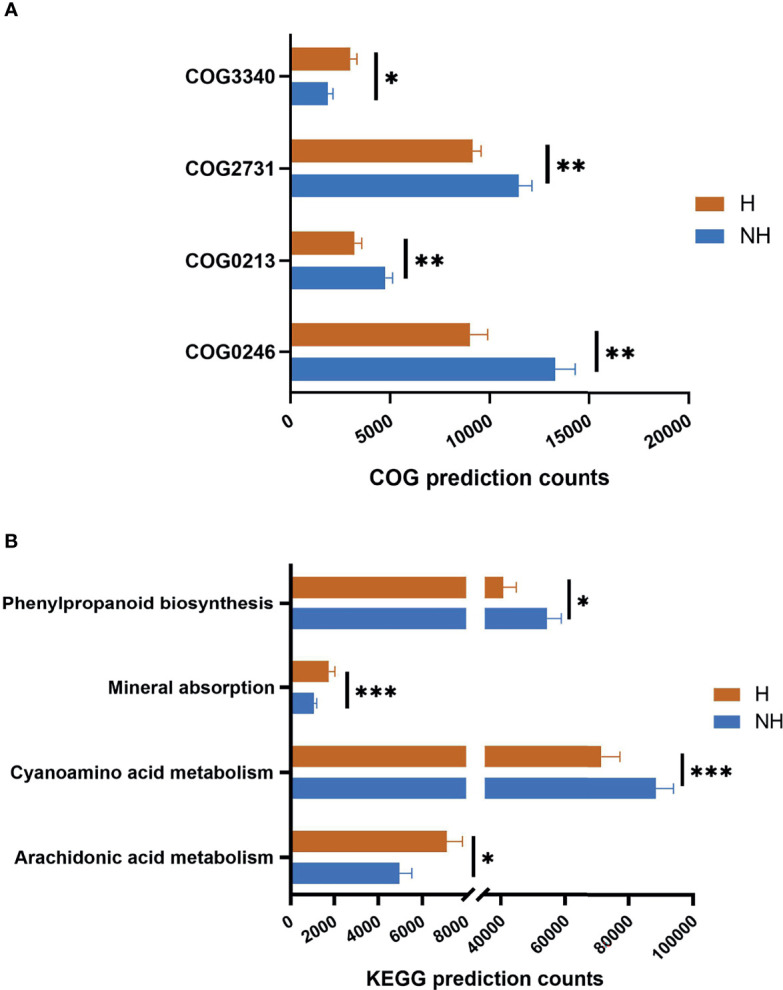
The distribution of functional categories. The distribution of **(A)** COG and **(B)** KEGG function with significant difference between two groups. The column length represents the corresponding samples in this metabolic pathway, and the error bar represents the standard error of abundance. *p < 0.05, **p < 0.01, ***p < 0.001.

As the composition of microbiota community altered, functional and metabolic changes could be predicted based on previous molecular studies (23, 24). Four Clusters of Orthologous Groups (COG) with *p* < 0.01 and four Kyoto Encyclopedia of Genes and Genome (KEGG) orthology with *p* < 0.05 were identified (**Figure 6**). The Protease E (COG3340, **Supplementary Table S1**) was significantly increased in the hematochezia group, while the β-galactosidase βsubunit (COG2731), the Mannitol-1-phosphate/altronate dehydrogenases (COG246), and the Thymidine phosphorylase (COG213) were downregulated (**Figure 6A**). Notably, mineral absorption and the metabolism of arachidonic acid (ARA) were significantly higher in the hematochezia group according to the annotation of the KEGG orthology (**Figure 6B**). It is known that ARA is not only an important composition of bacterial cell membrane but also interacts with mammalian cell to improve biosynthesis of inflammatory mediators prostaglandin (PGE2 and PGI2). Also, phenylpropanoid biosynthesis and cyanoaminoacid metabolism showed significant difference. The functional dysbiosis in CRP patients with hematochezia suggested an exacerbated inflammation state caused by rectal dysbiosis.

Functional analysis of rectal microbiota was also compared with healthy volunteers (**Supplementary Figure S6**). The Fe-S protein (COG1600), glutaredoxins (COG4545), and glutathione peroxidase (COG386) (25) were predicted to be significantly increased in both of the CRP groups. Mineral absorption and ARA metabolism in healthy controls were further reduced, compared with samples from CRP patients.

## Discussion

Rectal bleeding is the most common symptom of chronic radiation proctitis (CRP) that lays heavy medical burden to the patients. In this pilot study, we took gut microbiota samples from CRP patients under a proctoscope and revealed an altered microbial ecosystem in CRP patients with hematochezia.

CRP patients are mostly outpatients in surgery clinic seeking for remission of rectal irritation and hematochezia. It is believed that the signs and symptoms of CRP are largely contributed by interstitial fibrosis and occlusive endovascular inflammation (5, 6). Proctoscope examination provides a clearer vision of rectal mucus in a relatively convenient and cost-effective way (1). It is suggested that regular proctoscope could be beneficial to patients with radiation proctitis (3). Here, we included patients with an array of criteria including patients’ observation of blood in stool, digital rectal examination (DRE), and doctors’ observation under a proctoscope. A systematic history collection with multiple categories is helpful for not only research purposes but also outpatient management.

Dysbiosis has been demonstrated to be radiation-induced enteric inflammatory, although most of the research focused on acute enteritis. In a previous study on irradiation-inferred microbiome, *Clostridium IV*, *Roseburia*, and *Phascolarctobacterium* (13) were enriched. In this study, we found that relative abundances of these bacterium were similar between H and NH groups, implying a specific bacterium involves in prolonged bleeding. To our knowledge, this is the first study digging into the association between radiation-induced dysbiosis and chronic hemorrhage. A limitation of this study is the lack of pre-radiotherapy microbiome data, which could provide the baseline individual variation for better causative analysis and facilitate identification of biomarker for prognosis of pelvic cancer radiotherapy. A multicenter study with a larger sample size including male patients who survived other pelvic cancer is expected.

In this population of CRP patients, hematochezia is associated with an increased proportion of *Peptostreptococcaceae*, and *Peptostreptococcus* OTU45 is of most abundance in this family. High density of *Peptostreptococcaceae* was displayed in the intestine of humans with non-alcoholic fatty liver disease, suggesting the connection between *Peptostreptococcaceae* and impaired mucosal immune function (15). Dysregulated inflammation could be resulting from stress response. Gao et al. utilized a chronic stress mouse model to investigate the role of gut microbiota in stress-induced inflammatory bowel disease (IBD). They found that *Peptostreptococcaceae* were one of the inflammation-promoting OTUs and could be eliminated by antibiotics (26), which had also been considered as radiation response modifier. Clathrin adapter AP-1B is the epithelium-specific basolateral targeting factor that polarizes the epithelial cells, maintaining homeostasis of the gastrointestinal immune system. AP-1B was demonstrated to be the key factor of *Peptostreptococcaceae* abundance, which was significantly increased in *Aplm2* ^-/-^ colitis mice, but the detailed mechanism remains unclear (27). These lines of evidence suggest that *Peptostreptococcaceae* is highly associated with gastrointestinal inflammation, which may subsequently influence epithelial cell repair capacity or proliferation rate.

Diminished composition of *Lachnospiraceae* is also known to be related to gastrointestinal inflammation. In patients with IBD and acute colitis, *Lachnospiraceae, Ruminococcus* spp., *Faecalibacterium* spp., and *Roseburia* spp. were consistently depleted (28), suggesting that they are crucial gastrointestinal probiotics. Here,we observed that *Lachnospiraceae* OTU212 in the hematochezia group was significantly reduced, compared to that in the non-hematochezia group. Our LDA result also showed that genus *Lachnospiraceae*_FCS20 was higher in healthy controls, compared with CRP patients. Interestingly, *Lachnospiraceae* bacterium may contribute to the development of diabetes in obese mice (29) and calorie restriction resulted in lower *Lachnospiraceae* proportion (30). CRP with prolonged hematochezia and radiation enteritis patients have a relatively thin physique. However, the causation of specific *Lachnospiraceae* insufficiency and the decrease of body weight/mass index or diet intake remain to be elucidated.

Microbiota produce vitamins, energy sources, and amino acids by degradation and processing of diet-derived substrates. In this study, the differential mineral absorption, ARA metabolism, and Protease E between H and NH groups were predicted by PICRUSt. One possible explanation is that the micro-environment with continuous bleeding favors the high-metabolic-activity bacterium. The Grxs, FeS, and GPx upregulated in CRP patients are key regulators of cell redox and electron transport that are involved in various processes of biosynthesis and detoxification (31). Glutaredoxins (Grxs)-ligated Fe-S cluster participates in oxidative signaling (32) and haem synthesis (33), which may be compensatory to the prolonged anemia. The Grxs system also acts as an efficient electron donor to plasma glutathione peroxidase (GPx) (25). GPx is known as one of the major enzymes that prevent oxidative stress by catalyzing glutathione (GSH) into glutathione disulfide (GSSG). The increase of Grxs, FeS, and GPx may result from a high oxidative micro-environment after irradiation. In addition, GPx together with ARA enriched in the hematochezia group is capable of regulating prostaglandin PGE2 and PGI2, which promote gut inflammation (34). A prospective study of ARA and/or GPx effect on inflammation and hemorrhage in a post-irradiation model is expected in the future.

The dysbiosis could be related to the change of gene expression in glandular and/or epithelial cells induced by irradiation and inflammation (35). Thus, a combination of larger-scale metagenomic data, transcriptome profiles, and proteomic profile of an inflammatory factor study is expected to provide a comprehensive view of post-irradiation gut–microbiome interaction (36). In summary, this is a pilot study revealing the altered gut microbiota in CRP with hematochezia, providing a fundamental evidence for developing and evaluating treatments for CRP.

## Data Availability Statement

The datasets presented in this study can be found in online repositories. The names of the repository/repositories and accession number(s) can be found at: https://www.ncbi.nlm.nih.gov/genbank/, PRJNA682045.

## Ethics Statement

The studies involving human participants were reviewed and approved by the Ethics Board at the Affiliated TCM Hospital of Guangzhou Medical University. The patients/participants provided their written informed consent to participate in this study.

## Author Contributions

JL and LL conceived the study. LL contributed to the literature search, preparation of figures and panels, and writing of the manuscript. CC, BC, and XL contributed to sample collection clinical data analysis. CD contributed to programing data analysis. All authors have seen and agreed on the final submitted version of the manuscript.

## Funding

This article is an independent research funded by the the Affiliated TCM Hospital of Guangzhou Medical University (2018A01) and Guangdong Provincial Administration of Traditional Chinese Medicine (20211293).

## Conflict of Interest

The authors declare that the research was conducted in the absence of any commercial or financial relationships that could be construed as a potential conflict of interest.

## Publisher’s Note

All claims expressed in this article are solely those of the authors and do not necessarily represent those of their affiliated organizations, or those of the publisher, the editors and the reviewers. Any product that may be evaluated in this article, or claim that may be made by its manufacturer, is not guaranteed or endorsed by the publisher.
