# Supplementary material for: Altered Gut Microbiota Associated With Hemorrhage in Chronic Radiation Proctitis
Source: Front Oncol. 2021 Oct 15;11:637265. doi: 10.3389/fonc.2021.637265 (PMC8554627; doi:10.3389/fonc.2021.637265)
Supplement: Supplementary file 1 [file DataSheet_1.docx]

**Supplementary figures**


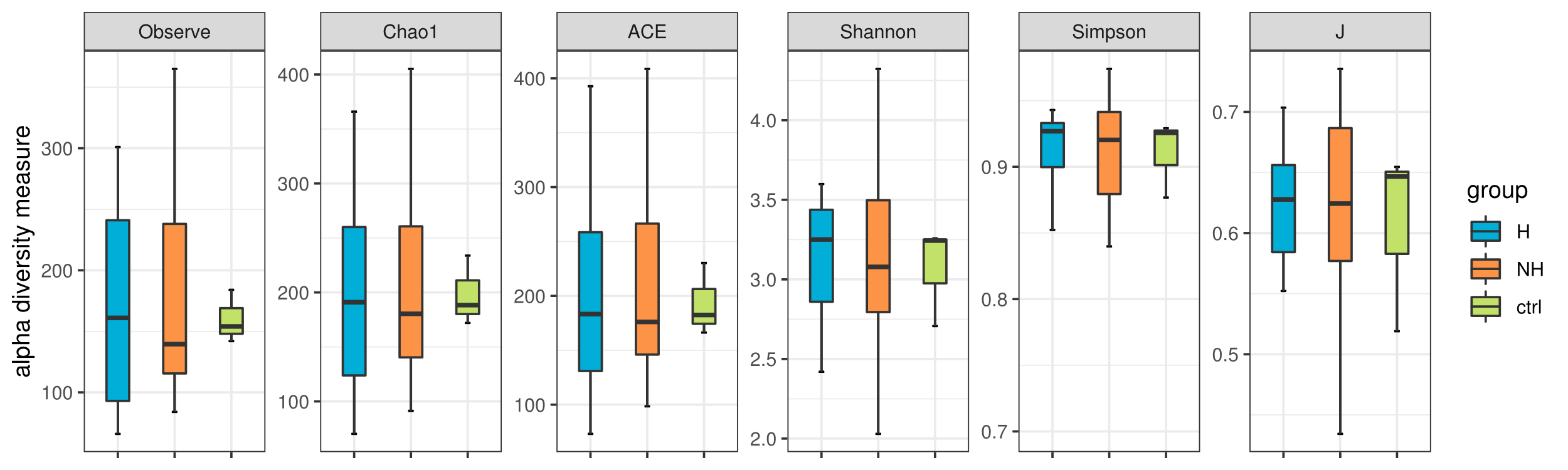


**Figure S1. Summary of α-diversity measurements** among heamafecia, non-heamafecia and healthy controls.

a
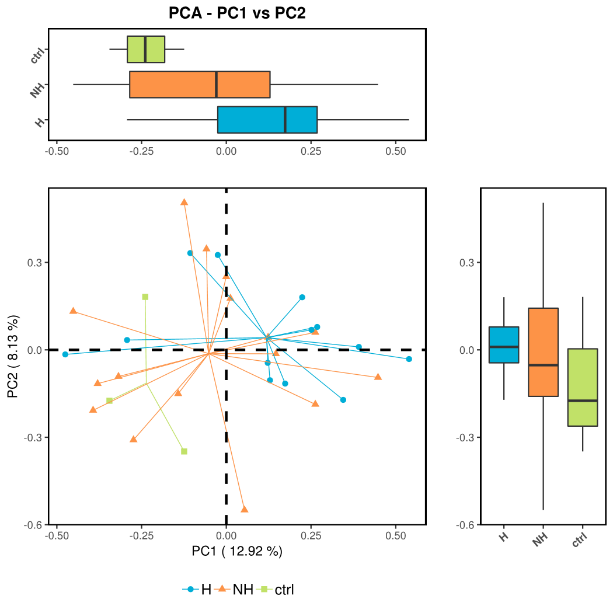
b
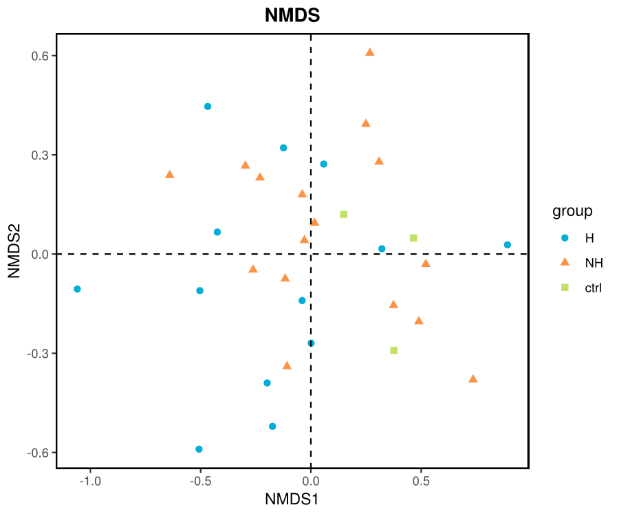


**Figure S2. Ordination plots** with (a) Principle Component Analysis (PCA) and (b) Non-Metric Multi-Dimensional Scaling (NMDS).

a
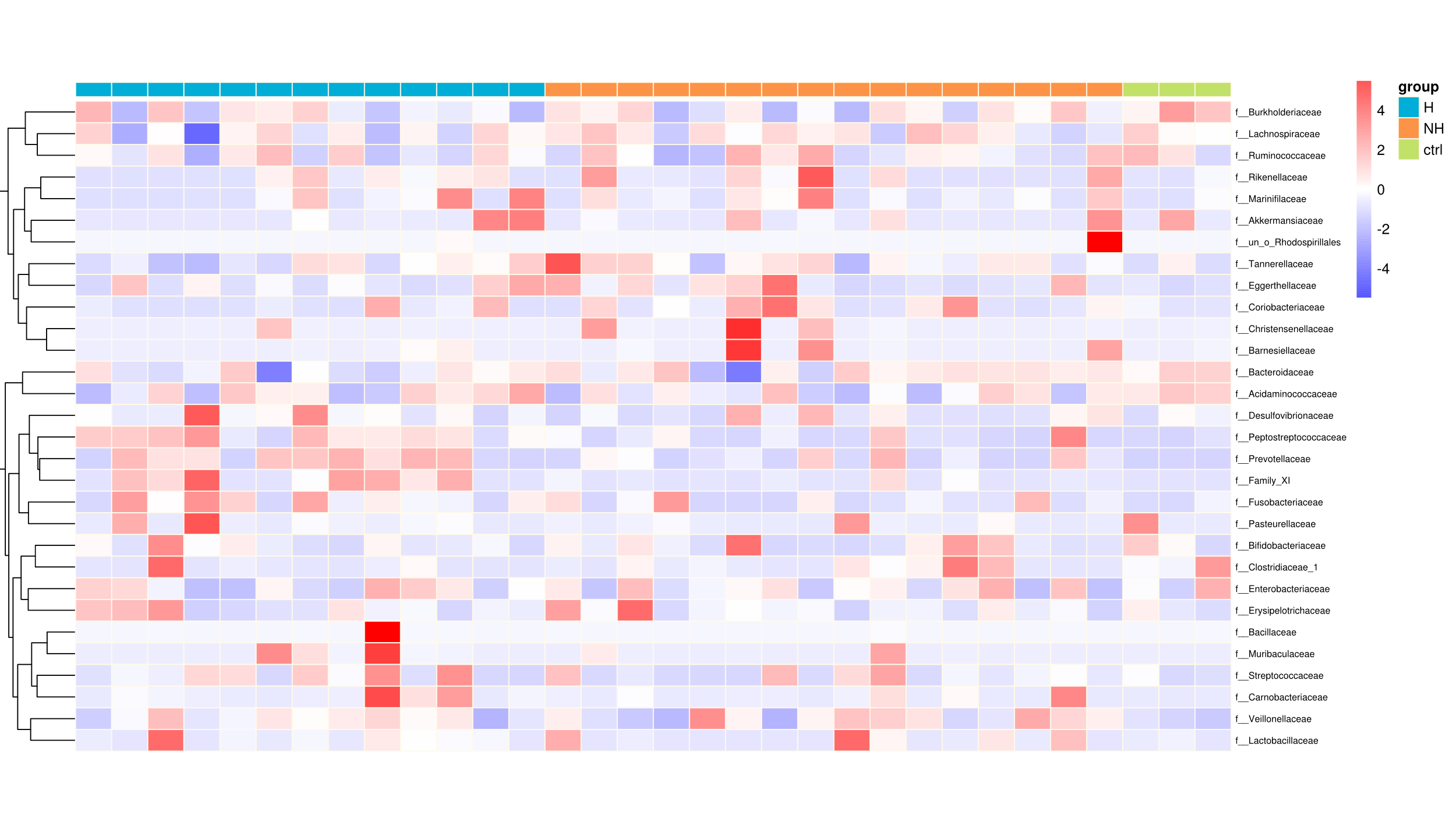


b
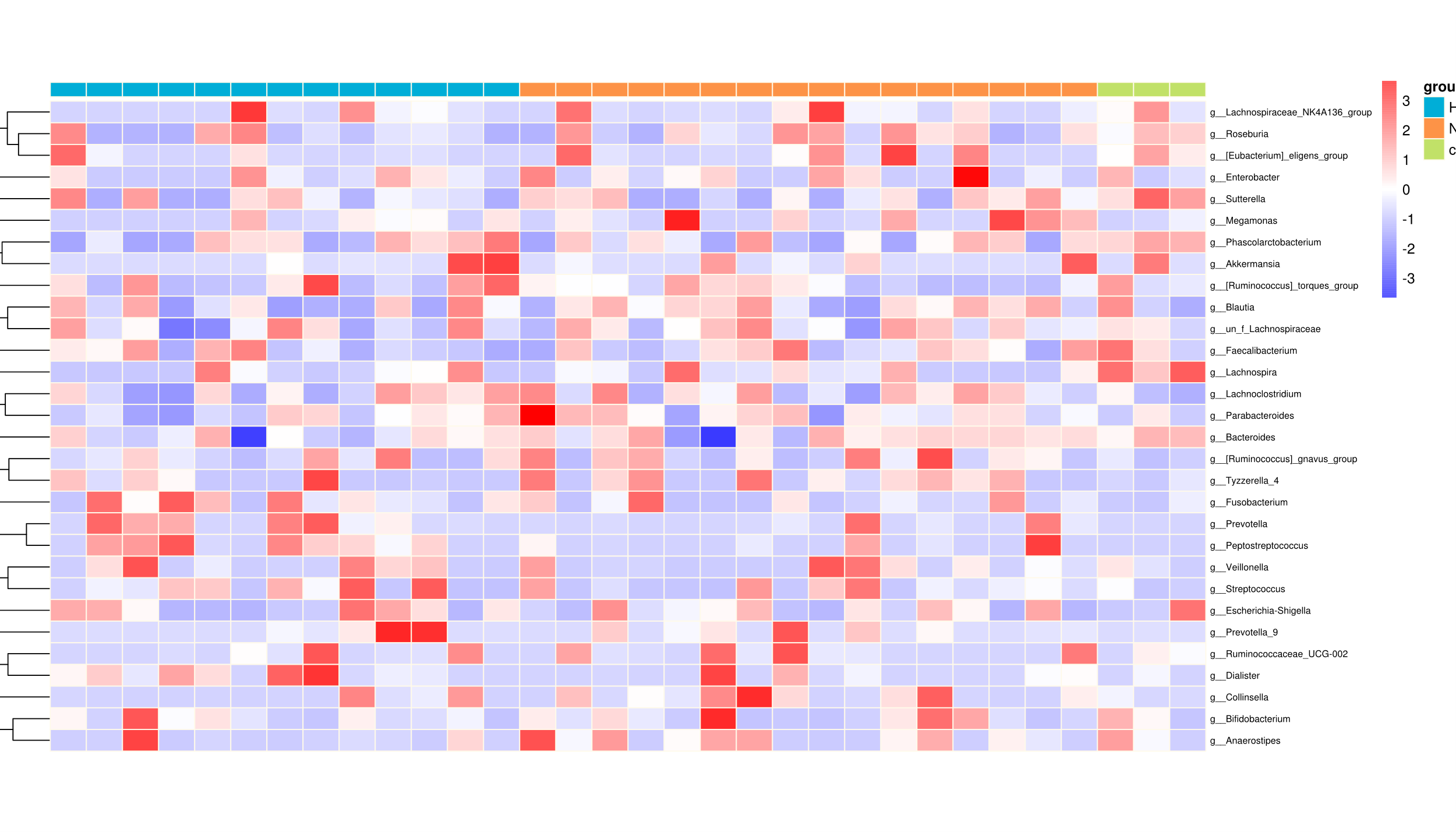


c
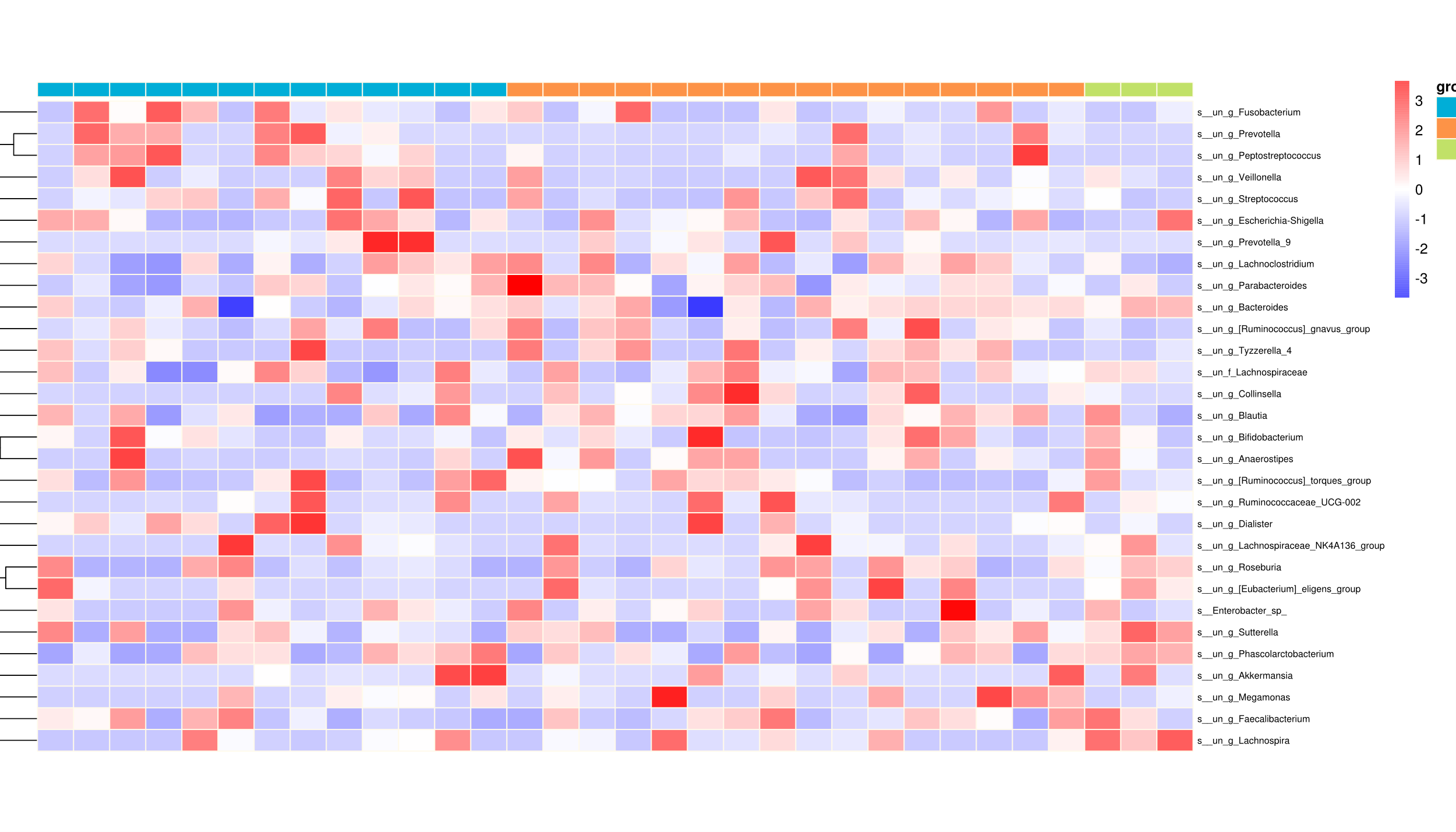


**Figure S3. Heatmap indicating the different abundance** of (a) families, (b) genera and (c) species between H, NH and healthy control groups of the microbiota.


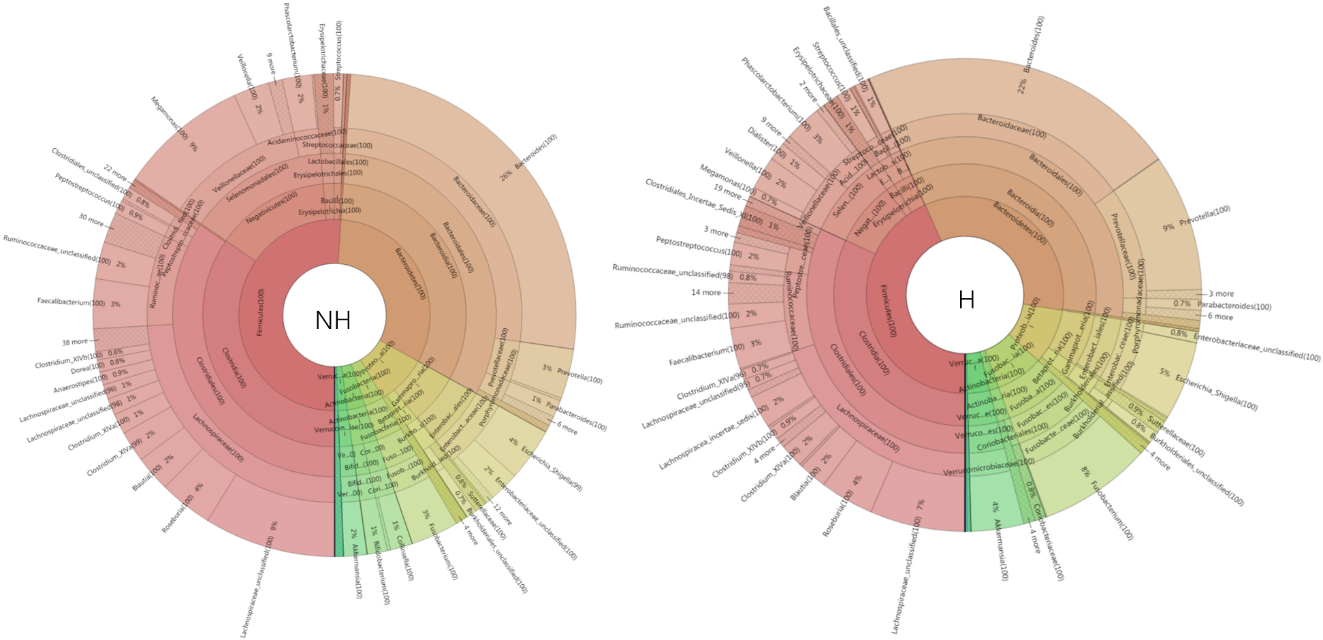


**Figure S4. Krona visualization showing microbiome composition of gut microbiota in each group.**


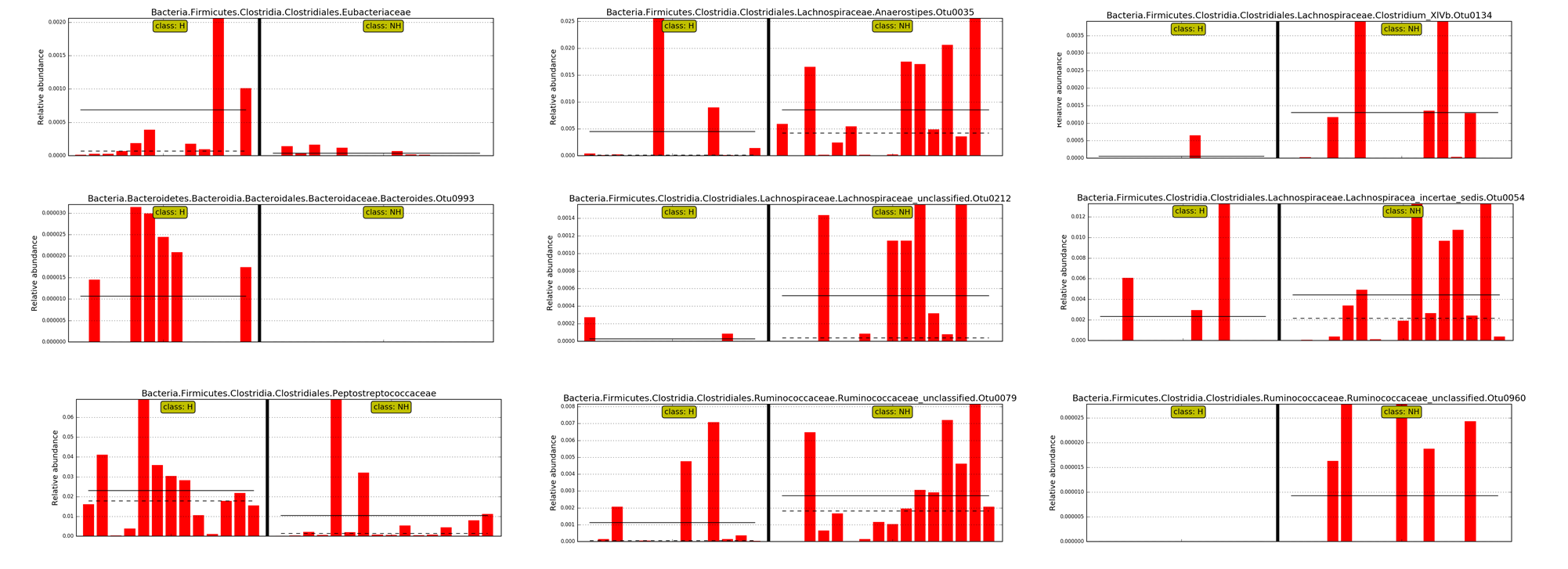


**Figure S5. Summary of LefSe analysis.** Solids line indicate mean; dot lines indicate SD.

a
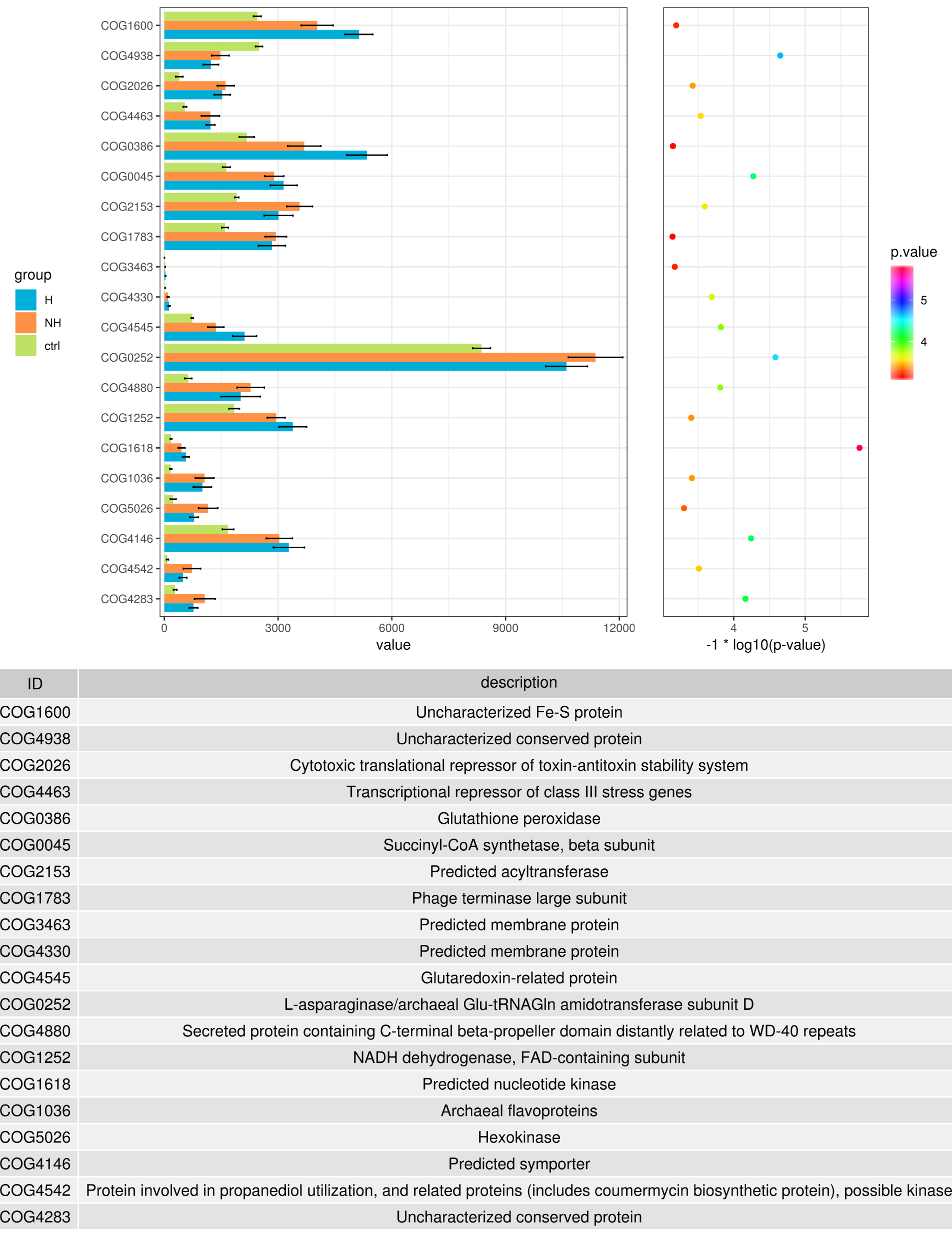
b


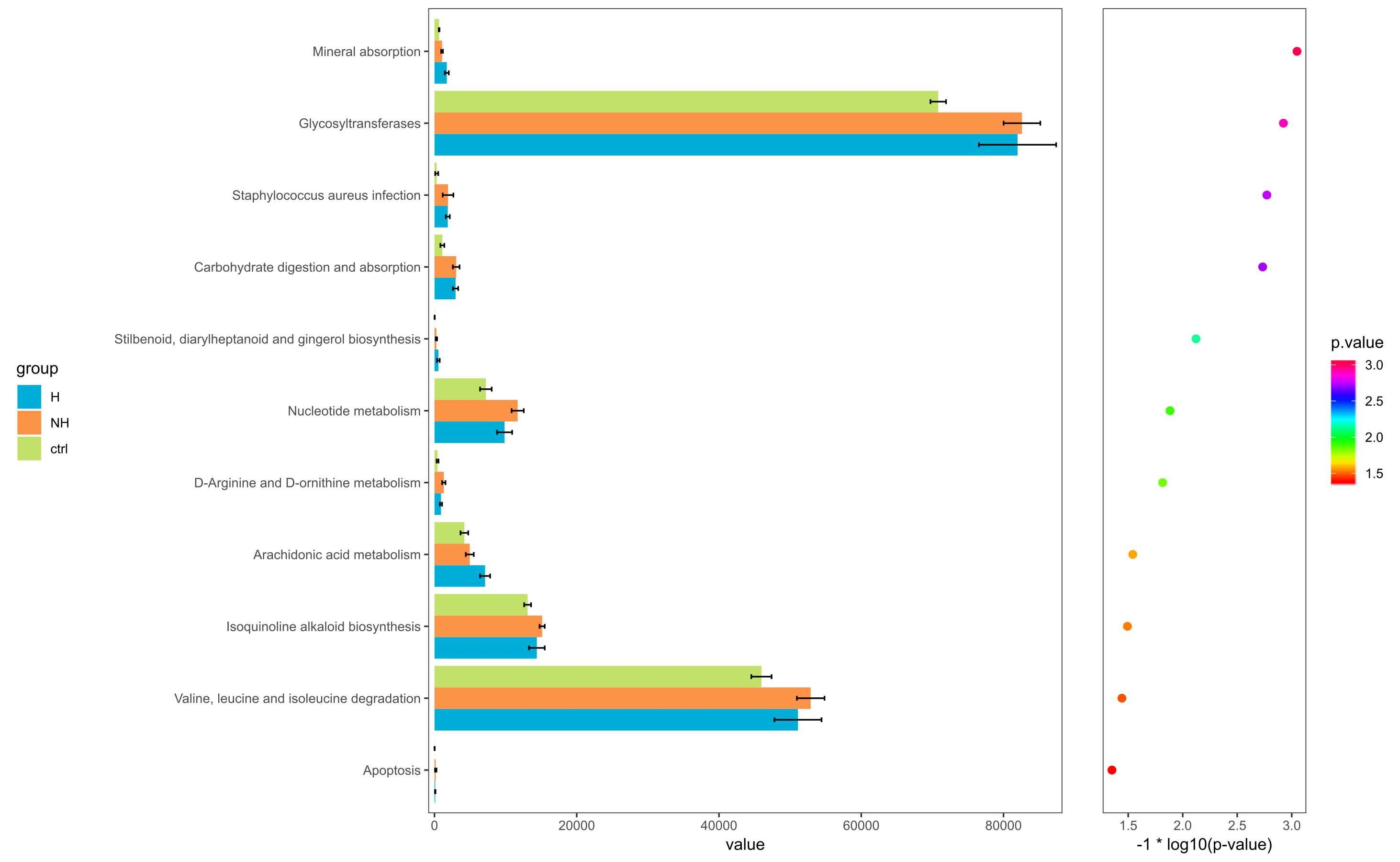


**Figure S6. The distribution of COG (a) and KEGG (b) functional categories differentiated significantly among heamafecia, non-heamafecia and healthy controls**. Dots on the right are FDR corrected p values.

**Supplementary table**

**Table S1. Description of identified COG pathways.**

| COG ID | Description |
| --- | --- |
| COG1600 | Uncharacterized Fe-S protein |
| COG4938 | Uncharacterized conserved protein |
| COG2026 | Cytotoxic translational repressor of toxin-antitoxin stability system |
| COG4463 | Transcriptional repressor of class III stress genes |
| COG0386 | Glutathione peroxidase |
| COG0045 | Succinyl-CoA synthetase, beta subunit |
| COG2153 | Predicted acyltransferase |
| COG1783 | Phage terminase large subunit |
| COG3463 | Predicted membrane protein |
| COG4330 | Predicted membrane protein |
| COG4545 | Glutaredoxin related protein |
| COG0252 | L-asparaginase/archaeal Glu-tRNAGln aminotransferase subunit D |
| COG4880 | Secreted protein containing C-terminal beta propeller domain distantly related to WD-40 repeats |
| COG1252 | NADH dehydrogenase, FAD-containing subunit |
| COG1618 | Predicted nucleotide kinase |
| COG1036 | Archaeal flavoproteins |
| COG5026 | Hexokinase |
| COG4146 | Predicted symporter |
| COG4542 | Protein involved in propanediol utilization, and related proteins (includes coumermycin biosynthetic protein), possible kinase |
| COG4283 | Uncharacterized conserved protein |
